# PAP900: A dataset of semantic relationships between affective words in Portuguese

**DOI:** 10.1016/j.dib.2025.111726

**Published:** 2025-05-30

**Authors:** André Fernandes dos Santos, José Paulo Leal, Rui Alexandre Alves, Teresa Jacques

**Affiliations:** a CRACS & INESC Tec LA / Faculty of Sciences, University of Porto, Portugal; b Faculty of Psychology and Education Sciences, University of Porto, Portugal

**Keywords:** Portuguese language, Semantic similarity, Semantic relatedness

## Abstract

The PAP900 dataset centers on the semantic relationship between affective words in Portuguese. It contains 900 word pairs, each annotated by at least 30 human raters for both semantic similarity and semantic relatedness. In addition to the semantic ratings, the dataset includes the word categorization used to build the word pairs and detailed sociodemographic information about annotators, enabling the analysis of the influence of personal factors on the perception of semantic relationships. Furthermore, this article describes in detail the dataset construction process, from word selection to agreement metrics.

Data was collected from Portuguese university psychology students, who completed two rounds of questionnaires. In the first round annotators were asked to rate word pairs on either semantic similarity or relatedness. The second round switched the relation type for most annotators, with a small percentage being asked to repeat the same relation. The instructions given emphasized the differences between semantic relatedness and semantic similarity, and provided examples of expected ratings of both.

There are few semantic relations datasets in Portuguese, and none focusing on affective words. PAP900 is distributed in distinct formats to be easy to use for both researchers just looking for the final averaged values and for researchers looking to take advantage of the individual ratings, the word categorization and the annotator data. This dataset is a valuable resource for researchers in computational linguistics, natural language processing, psychology, and cognitive science.

Specifications Table

**Table:** 

Subject	Computer Science - Artificial Intelligence
Specific subject area	Human-reported values for semantic similarity and relatedness between Portuguese affective words.
Type of data	Tables. Raw, Analyzed, Filtered.
Data collection	University psychology students were asked to rate the semantic similarity and semantic relatedness of pairs of affective words in Portuguese. The students answered by filling out two online questionnaires, presented approximately one month apart. Questionnaires asked for the rating of 200 pairs of words each, randomly sampled from a set of 900 pairs of words. Questionnaires also included questions regarding the sociodemographic data of the annotators. Word order and pair order were randomized in each questionnaire. Outlier student responses were excluded based on their low correlation with the average values reported by all students.
Data source location	Institution: University of Porto City/Town/Region: Porto Country: Portugal
Data accessibility	Repository name: Mendeley Data Data identification number: 10.17632/5mhxtv8pn2.2 Direct URL to data: https://data.mendeley.com/datasets/5mhxtv8pn2/2
Related research article	none

## 1 Value of the Data

• The dataset establishes a gold standard for Portuguese-language semantic relations of affective words, a field that is significantly under-represented, with only a few existing datasets for Portuguese semantic relations and affective words, and none specifically for Portuguese affective words.
• Researchers in computational linguistics and natural language processing can use this data to train and evaluate semantic measure algorithms or language models, and in research involving sentiment analysis or emotional word processing. Given its focus on emotions, psychologists and cognitive scientists can also leverage this data to analyze affective word processing, or how emotions can be clustered and organized. Unlike most existing datasets, PAP900 includes separate values for semantic similarity and relatedness, enabling researchers to compare how human annotators perceive these concepts differently. These distinct values facilitate research into the connection between semantic relatedness and similarity. Researchers can also leverage this information in studies focusing specifically on either semantic relation.
• The detailed sociodemographic data and raw individual scores for each annotator allow for the analysis of how personal characteristics (e.g. age, gender) can influence judgements of semantic relationships.
• The comprehensive documentation of the methodology used in constructing the dataset, including word pair selection, annotator assignment, annotator instructions, agreement calculations, and annotator raw data, can be used to replicate this study, to develop comparable studies, or as an overall reference for best practices in semantic relations dataset construction.
• The dataset is freely available and explicitly released under a permissive license (CC BY-ND 4.0), allowing it to be reused and shared with very few restrictions.

## 2 Background

Semantic similarity and relatedness metrics are essential for numerous tasks in computational linguistics and NLP. Evaluating these algorithms typically hinges on having a baseline or benchmark. However, creating gold standards for semantic relations (SR) is particularly challenging due to the inherent subjectivity of semantic distance, the complexity of measuring it objectively, and the manual labour required to complete this task. SR datasets play an important role in artificial intelligence and natural language processing tasks such as development and evaluation of semantic measures and word embeddings, semantic search and information retrieval, spelling correction and machine translation. SR datasets are typically composed of pairs of units of language (e.g. texts, sentences, concepts) and human-attributed numeric values representing the strength of the semantic connections between them. Hadj Taieb et al. [2] and Chandrasekaran and Mago [3] provide overviews over the semantic relatedness and similarity datasets field.

SRs are often divided in two categories: semantic similarity, the degree to which elements are taxonomically close (i.e. the properties they share and how their meaning overlaps), and semantic relatedness, which includes other types of semantic connections (e.g. when elements are frequently used together, when they are opposites, when one element is part of the other).

Affective words convey feelings, emotions and attitudes [4]. Individuals differ in emotional granularity [5]. People with high emotional granularity tend to have a nuanced vocabulary that helps to distinguish emotions [6]. The human emotional experience is so vast that Brown [7] has identified 87 different human emotions and experiences, which can be tagged with distinct affective words. This set of words has also been included in PAP900.

## 3 Data Description

PAP900 [1] contains 900 pairs of affective words in Portuguese and the corresponding scores for semantic similarity and relatedness. These scores were derived by averaging ratings from approximately 30 annotators per pair, with over 200 annotators contributing in total. Additionally, PAP900includes sociodemographic information and the raw individual ratings of the annotators.

PAP900 is made available in 3 different formats, **average** (final averaged values), **raw** (all the raw anonymized data), and **curated-matrix** (individual scores for each pair). Detailed descriptions follow:

• **average** includes only the final averaged values for relatedness and similarity for each pair of words. It is composed of two files:
  ○ *relatedness.csv*: average values reported for semantic relatedness. Contains the following fields:
    ■ **Term1:** first word of the pair
    ■ **Term2**: second word of the pair
    ■ **Value**: decimal number representing the averaged semantic relatedness of the pair
  ○ *similarity.csv*: average values reported for semantic similarity: Contains the following fields:
    ■ **Term1:** first word of **the** pair
    ■ **Term2**: second word of the pair
    ■ **Value**: decimal number representing the averaged semantic similarity of the pair
• **raw** includes all the raw data, including the list of words adapted and translated from Atlas of the Heart and LexEmo.pt, anonymized annotator sociodemographic information and individual responses. It is composed of 3 files plus one file per annotator:
  ○ *words.csv*: words and corresponding categories used to generate the pairs (more information on Section *Word Pair Selection*). Contains the following fields:
    ■ **AotH_word**: original Atlas of the Heart word in English
    ■ **AotH_category**: original Atlas of the **Heart** category
    ■ **word_pt**: translation to Portuguese of **AotH**_word
    ■ **pt_adj_M**: conversion of word_pt to **adjective**, masculine form
    ■ **pt_adj_F**: conversion of word_pt to **adjective**, feminine form
    ■ **polarity, hyper_cat, super_cat** and **basic_cat**: *word_pt* categorization according to LexEmo.pt
  ○ *info.csv*: information *about* the annotators. Contains the following fields:
    ■ **annoId:** random ID assigned to the student
    ■ **studYear:** number of year of the student in the university
    ■ **uc:** course unit in which context the student answered the questionnaire
    ■ **nativeLanguage:** native language of the student (ISO 639)
    ■ **bilingual:** whether the student is bilingual
    ■ **otherLangs:** other languages spoken by the student (ISO **639**)
    ■ **gender:** gender of the student
    ■ **ageBracket:** age of the student
  ○ *outliers.csv*: list of IDs of annotators whose responses were considered outliers
  ○ {XYZ}-{Vn}-{MT}-{F2M}_pairs.csv: pairs and values for annotator XYZ.
    ■ File name fields:
      • **XYZ**: annotator ID
      • **Vn**: form version (V1/V2)
      • **RT**: relation type (similarity/**relatedness**)
      • **F2M**: form V2 type (switch/**repeat**). Only for V2 responses
    ■ File content fields:
      • **Term1**: first word of the pair
      • **Term2**: second word of the pair
      • **Value**: semantic relation value
      • **NormalizedTerm1** and **NormalizedTerm2**: normalized versions of **Term1** and **Term2**. Normalisation consists of converting words to the masculine gender and sorting the words in the pair alphabetically.
• **curated-matrix** includes the annotator individual values, but gathered on two simple matrices (one for each relation type), with outliers removed. It is composed of two files:
  ○ relatedness.csv: Annotator responses for semantic relatedness. Contains the following fields:
    ■ **Term1**: first word of the pair
    ■ **Term2**: second word of the pair
    ■ **AnnoID1** to **AnnoIDN**: semantic **relatedness** value for the pair, as reported by each annotator
      • the columns are named after the annotator ID
  ○ similarity.csv: Annotator responses for semantic similarity. Contains the following fields:
    ■ **Term1**: first word of the pair
    ■ **Term2**: second word of the pair
    ■ **AnnoID1** to **AnnoIDN**: semantic similarity value for the pair, as reported by each annotator. The columns are named after the annotator ID

Listing 1, Listing 2, Listing 3 present excerpts of files from the PAP900 dataset.

**Listing 1:** Excerpt from *average/relatedness.csv.*

**Term1,**	**Term2,**	**Value**
alegre,	alegre,	4.000
alegre,	apavorado,	0.525
alegre,	arrogante,	0.395
alegre,	calmo,	1.270
alegre,	ciumento,	0.184
(...)

**Listing 2:** Excerpt from curated-matrix/similarity.csv.

**Term1,**	**Term2,**	**0eD6A,**	**29Hgl,**	**2AYpo,**	**2m1cv,**	**2mCuF,**	**2SIcT,**	**(...)**
alegre,	alegre,	,	,	,	,	4,	4,	
alegre,	apavorado,	,	0,	,	,	,	1,	
alegre,	arrogante,	0,	,	,	0,	,	,	
alegre,	calmo,	1,	,	1,	3,	,	,	
alegre,	ciumento,	,	1,	,	1,	,	,	
(...)

**Listing 3:** Excerpt from *raw/info.csv.*

**annoId,**	**studYear,**	**uc,**	**nativeLanguage,**	**bilingual,**	**otherLangs,**	**gender,**	**ageBracket**
DvNPR,	1,	PPL,	pt,	1,	fr;en,	F,	18-19
FoDnI,	2,	PPL,	pt,	0,	,	F,	18-19
nBCpV,	1,	PPL,	pt,	1,	,	F,	18-19
qm12C,	1,	PPL,	pt,	0,	en,	F,	18-19
B3C6R,	1,	PPL,	pt,	0,	,	F,	18-19
(...)							

166 students replied to both versions of the questionnaire and 47 replied only to the first questionnaire. The total number of responses (V1+V2) is 379. We excluded 9 students with outlier responses (those whose Pearson's correlation with average values was more than 3 standard deviations below the average correlation). This resulted in 204 valid annotators and 367 valid responses. The final dataset has 900 pairs of words, each rated by an average of 38 annotators. No pair was rated by less than 30 people.

**Fig. 1:**
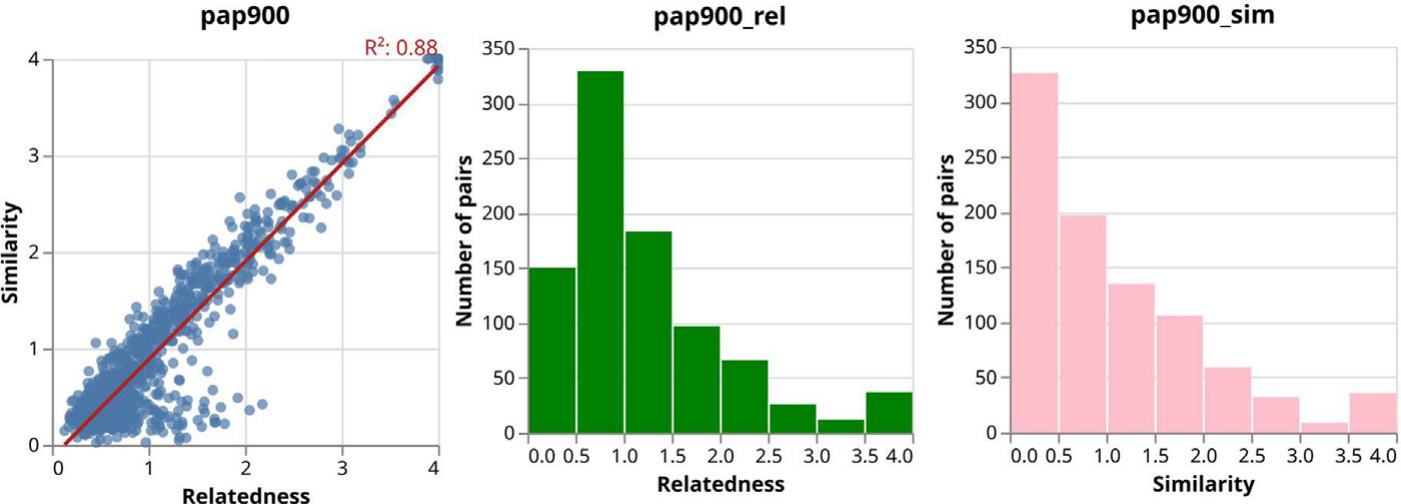
Relatedness and similarity values in PAP900. **a)** Pair relatedness vs pair similarity. **b)** Pair relatedness value distribution. **c)** Pair similarity value distribution.

The gathered responses reveal a stronger agreement on similarity. The average correlation with consensus (AMIAA, see Section *Annotator Agreement Calculations*) was 0.757 for similarity scores and 0.675 for relatedness scores. Factors such as annotators' age, gender, or multilingualism did not appear to influence their evaluation of semantic relationships, as the AMIAA values remained relatively close^1^. Fig. 1 presents multiple views of the distribution of values for relatedness and similarity on PAP900. Fig. 1a**)**, in particular, shows a general correlation between the two metrics. However, a notable exception includes pairs with low similarity but higher relatedness scores. Manual inspection reveals that these pairs often have opposite polarities, such as *(``contente'', ``triste''), (``stressado'', ``tranquilo'')*, and *(``alegre'', ``desolado'')*.

## 4 Experimental Design, Materials and Methods

### 4.1 Word pair selection

In our selection process for words and word pairs we had two main goals: to ensure a wide selection of affective words, and to generate pairs with a good distribution of expected similarity and relatedness values.

We started with the list proposed by Atlas of the Heart (AotH), containing *87 human emotions and experiences* [7] (mostly nouns), which we translated to Portuguese. For each word we obtained, whenever possible, the corresponding categorization from LexEmo.pt [8], a Portuguese affective words database in which words are categorized according to their polarity, hyper category, super category and base category. This resulted in 69 Portuguese words along with their categorization.

Each Portuguese word was then converted into its adjective form. Using the same part of speech of all words makes them easier to compare against each other. Adjectives are also more common in the emotional lexicon, and arguably facilitates annotation by making ratings more intuitive. The dataset includes the original AotH words and their categorization, along with the corresponding Portuguese nouns, adjectives, and LexEmo.pt categorizations. However, the final list does not maintain a one-to-one correspondence with the 87 AotH emotions due to factors such as multiple possible translations into Portuguese and the absence of certain words in LexEmo.pt.

**Fig. 2:**
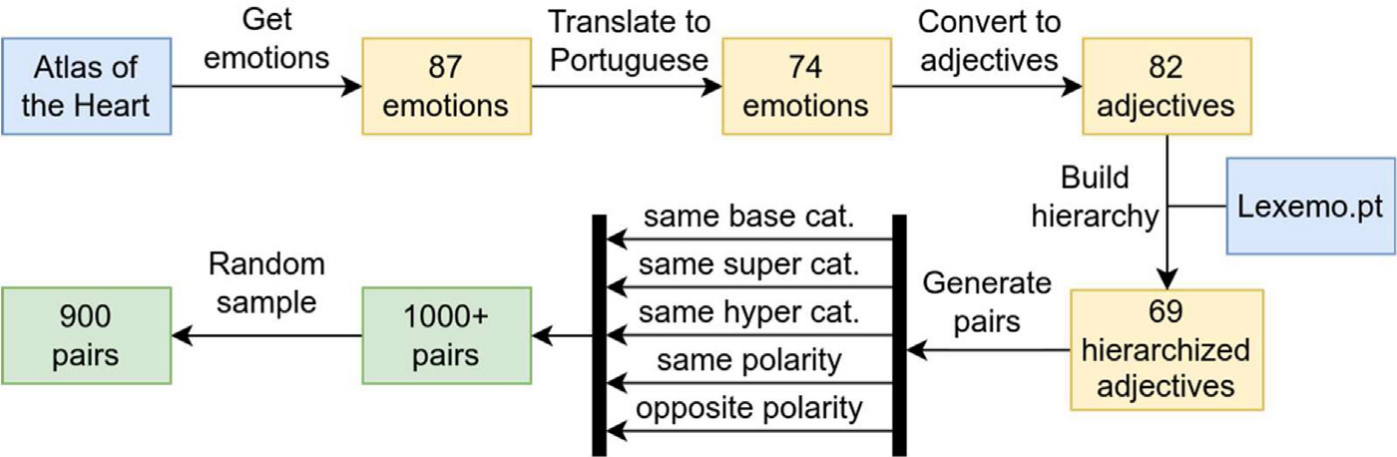
Word selection and pair generation processes in PAP900.

We then generated pairs of words as follows: first, we randomly selected to form pairs words with different polarity; then words sharing only their polarity (but different hyper, super and basic categories); then words sharing their hyper category, but different lower category levels; and so on, until we generated pairs sharing the same basic category. We generated more than 1000 pairs, and then randomly selected 900 to be used (a number we estimated would allow us to have around 30 annotators per pair). The distribution categories in these 900 pairs was the following: 448 pairs with different valence; 268 with the same valence but different categories; 89 with the same hyper category but different lower categories; 35 with the same super category but different base categories and 60 pairs with the same base category. Fig. 2 presents a diagram of this process.

For each annotator both versions of the questionnaire included 200 pairs of words randomly sampled from this list.

### 4.2 Annotator instructions

Listing 4 contains a translation to English of the instructions (originally in Portuguese) given to the annotators before being asked to rate the semantic relatedness of 200 pairs of words. Instructions for semantic similarity were identical.

**Listing 4:** English translation of the instructions given to annotators (relatedness version).

In the next sections of this form you will be asked to rate quantitatively, on a scale, the strength of the semantic relation between pairs of affective words. Before you start, please read carefully the instructions and examples provided. Please note that opposite words are frequently highly related.
For example, the words **modest** and **smart** seem mostly unrelated. **Conceal** and **disguise** however seem highly related. **Confident** is very related to itself. **Violent** and **peaceful**, while being opposite words, are frequently very related, just like **happiness** and **sadness**.
The question we are asking is: how related are the two words? Pairs of words not related should be rated with lower values, and pairs of words highly related with higher values.
• modest, smart: not related (1) • conceal, disguise: vaguely related (2) • confident, confident: totally related (5) • violent, peaceful: very related (4) • happiness, sadness: totally related (5)

At the end of the questionnaire the annotators were also asked to report any unknown words they encountered, and pairs containing those words were removed from that annotator’s answers.

### 4.3 Annotator characterization

Our goal for this dataset was not to collect SR ratings from linguists or other language experts who might consider factors like etymology or lexicography. Instead, we aimed to capture the judgments of everyday speakers, as is common in non-specialized SR datasets. For logistic and research reasons, all our annotators were psychology students, who may have a greater-than-average understanding of emotions.

Annotators were gathered from students attending three different units from Psychology degrees from the University of Porto, Portugal. Students were mostly female (186 self-identified as women, 23 men and 4 other), the average age was 22.3 years (standard deviation 6.3 years) and the vast majority (97.6%) were native Portuguese speakers. All this and more sociodemographic data is included in the dataset.

### 4.4 Annotation setup

**Fig. 3:**
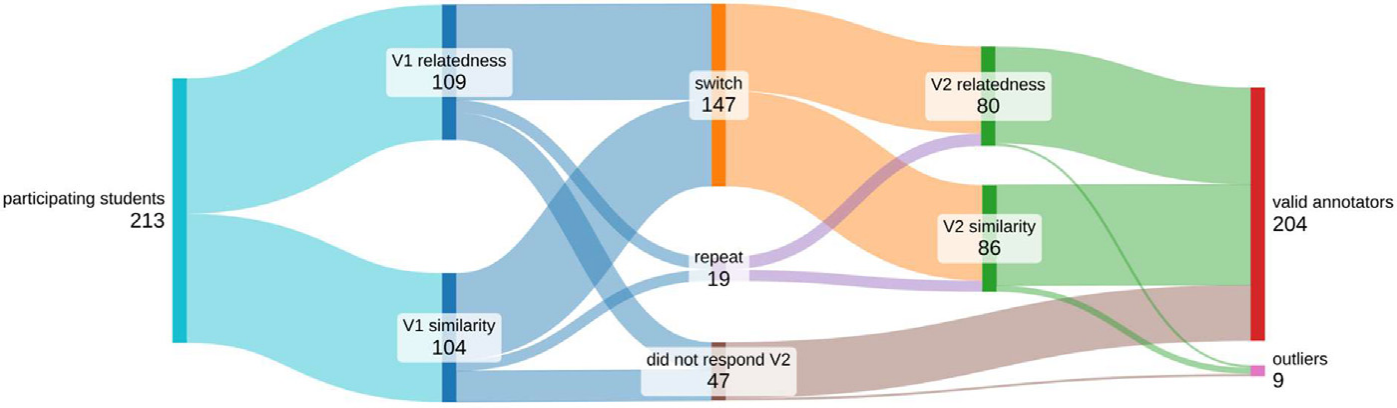
Annotator flow on the PAP900 construction process.

The answers were gathered via an online questionnaire where each student was asked to fill in some sociodemographic information and to rate 200 pairs of words. The students were asked to rate pairs of words in two different moments which were, on average, one month apart. Fig. 3 presents a flow diagram of the annotators pipeline.

On the first questionnaire 50% of the students were asked to rate the pairs of words according to their semantic relatedness, and 50% according to their semantic similarity. The questionnaire included instructions on the distinction between semantic similarity and relatedness as well as examples of word pairs and their plausible rating for each relation type (see Section 4.2). The order of the pairs and the order of the words in each pair were randomized. The gender of the words was matched to the gender reported by the student.

On the second questionnaire, 10% of the students were asked to repeat exactly the same exercise as before: rating the same pairs of words, according to the same relation type (form V2 mode *repeat*). The other 90% were asked to rate the same pairs, but switching the relation type (form V2 mode *switch*). So students who were first asked about relatedness were then asked to rate similarity, and vice-versa. For all students the order of the pairs and the order of the words in each pair in the second questionnaire were again randomized.

The responses to the second questionnaire of the 10% of students asked to repeat the rating of the same pairs and same relation type were used to calculate intra annotator agreement. These responses are available in the *raw* format, but were excluded from the averaged values in the *average* and *curated-matrix* to avoid skewing the results.

### 4.5 Outlier detection

For outlier response detection we used two approaches: time spent filling out the form by each annotator, and the correlation of the annotator ratings with the average of the values reported by the other annotators.

### 4.6 Time spent filling out the form

Because the first questionnaire was only created for each user when they first tried to access it, we were able to calculate the difference between the form creation and submission. A limitation of this approach is that some students clearly triggered the form creation but then abandoned the task, only to resume it hours or days later. The second questionnaire was triggered by the submission of the first, so we could not use the same approach.

Forms submitted too fast would be a good indicator of an annotator not very compromised with accuracy and probably providing random answers. However, the fastest responses we obtained were around 10 minutes, which is not significantly lower than the fastest achieved by the authors (15 minutes), so no annotators were excluded this way.

### 4.7 Response correlation with average

For each response, we calculated the Person correlation coefficient with the average values of the other responses. Then we calculated the average correlation coefficient. Responses whose correlation with average were more than one standard deviation below the mean resulted in the exclusion of both responses (first and second questionnaire) of corresponding annotator.

This resulted in the exclusion of 9 annotators and 12 responses.

### 4.8 Annotator agreement calculations

Datasets described in the literature frequently use different metrics to report their inter and intra annotator agreements. Frequently Pearson’s or Spearman’s correlation coefficient formulas are used, but what they are applied to varies widely.

Vulić et al [9] propose the metrics average pairwise inter-annotator agreement (APIAA) and average mean inter-annotator agreement (AMIAA), defined as follows:

$$1)\;\text{APIA}A_{\rho }=\frac{2\sum_{i<j}\rho \left(s_{i},s_{j}\right)}{N\left(N-1\right)}\;\;2)\;\text{AMIA}A_{\rho }=\frac{\sum_{i}\rho \left(s_{i},\mu_{i}\right)}{N},\;\text{where}:\mu_{i}=\frac{\sum_{j,j\neq i}s_{j}}{N-1}$$

with *ρ(s_i,_ s_j_)* being the Spearman’s correlation between annotators *i* and *j* and *N* the number of annotators. AMIAA is the metric closest to how semantic measure algorithms are evaluated (correlation between the predictions of the algorithm and the average values of the dataset).

For PAP900 we calculated the same inter-annotator agreement metrics but using Pearson’s correlation instead of Spearman’s: APIAA*_r_* and AMIAA*_r_*. The AMIAA*_r_* obtained was 0.716, with a standard deviation of 0.159. For calculating the APIAA*_r_* we had one additional challenge. To calculate the correlation between the scores of two annotators would require them to have annotated the same pairs. But the 200 pairs of words attributed to each annotator were randomly sampled from the larger 900 pairs collection, so each annotator got their own set of pairs, with varying overlap with other annotators. We decided to calculate the intersection between the pairs annotated by each two annotators. If the resulting list contained less than 10 pairs, it was discarded. We calculated the Pearson correlation for the resulting intersection lists of pairs, and in the end we obtained an APIAA*_r_* of 0.499, with a standard deviation of 0.225.

We calculated the intra annotator agreement by having a small group (19) of annotators repeating the exact same pairs of words and relation type on the second questionnaire. We measured the Pearson correlation between each annotator’s first and second questionnaire score. The average of these correlations was 0.667, with a standard deviation of 0.101.

## Limitations

Our annotators were all students of psychology at the University of Porto, Portugal, and, as such, they are more homogenous than a random sample of a larger population. Most of them were women, below 30 years of age, most of them Portuguese and living in the same city.

The inter-annotator agreement values we obtained were lower than those reported in other datasets. Several factors likely contributed to this, with two standing out: the annotators' lack of expertise and the specific domain of the word pairs (emotions and feelings).

None of the annotators were experts in semantic relations. Despite our efforts to provide more detailed instructions than is typical for dataset construction, they remained brief, and no formal training was conducted. Investing additional time in training might have improved inter-annotator agreement. In specialized domains (e.g., medical or geographical datasets), annotator expertise likely enhances dataset quality; however, the objective of this dataset was to capture human perception of semantic connections between emotions. Excessive training could have introduced unintended bias.

The nature of the dataset's domain may also explain the lower inter-annotator agreement. Affective words, which represent abstract concepts and subjective feelings, are inherently harder to compare objectively than concrete entities like animals or objects.

## Ethics statement

All human annotators involved in this work have consented to the release of the produced individual label data. In addition, the annotators have been anonymized in the released datasets.

## CRediT Author Statement

**André Fernandes dos Santos:** Conceptualization, Methodology, Software, Validation, Formal analysis, Data curation, Writing - original draft, Visualization. **José Paulo Leal:** Conceptualization, Methodology, Software, Writing - Review & Editing, Supervision, Project administration, Funding acquisition. **Rui Alexandre Alves:** Conceptualization, Investigation, Resources; Writing - Review & Editing. **Teresa Jacques:** Conceptualization, Investigation, Resources; Writing - Review & Editing.

## Declaration of Competing Interest

The authors declare that they have no known competing financial interests or personal relationships that could have appeared to influence the work reported in this paper.

## Data Availability

Mendeley DataPAP900 (Original data).

## Footnotes

1 The code used to calculate these and more metrics can be found at https://github.com/andrefs/pap900-metrics.
